# The effectiveness of community-based rehabilitation as a strategy for improving quality of life and disaster resilience for children with disability in rural Zimbabwe

**DOI:** 10.4102/jamba.v10i1.442

**Published:** 2018-04-17

**Authors:** Pathias P. Bongo, Gladys Dziruni, Chipo Muzenda-Mudavanhu

**Affiliations:** 1 Department of Geography, Bindura University of Science Education, Zimbabwe; 2 World Vision Inc, Harare, Zimbabwe

## Abstract

The study aimed to investigate the effectiveness of the community-based rehabilitation (CBR) project in Ward 20 of Chipinge in Zimbabwe and ascertain the positive district changes in the quality of life and disaster resilience of children with disability. Effectiveness involved examining the role of the parents of children with disabilities and the general community in the CBR programme, the extent to which children living with disabilities (CWDs) have been empowered to live quality life and access basic social services and evaluate whether local resources and capacities were being utilised. Data were collected through key informant interviews, document analysis and focus group discussions. The CBR model borrows heavily from rights-based approaches to development. Its practical application is problematic because of difficulties in defining issues such as participation and the ability of developing and poor communities to generate resources for these programmes. The study found that factors that hinder the effectives of CBR programmes included continuous dependence on donor funding, lack of political will by government and local authorities to commit financial resources towards CBR implementation and unreliable referral systems for access of services for children with disability. Gaps identified include establishing appropriate context-specific strategies that suit developing countries. The government and local authorities should prioritise resource allocation for marginalised groups such as people with disabilities. Civil society should not be the major and only source of funding for CBR. Extensive consultations should be made to adapt the CBR model to the socio-economic context of developing countries. The referral system for access to services for CWDs should be strengthened.

## Introduction and background

People with disabilities have been defined as one of the largest minorities. Recent studies suggest that people with disabilities represent 10% – 12% of the world’s population (Busapathumrong, 2013; Jackson & Mupedziswa 1988). The United Nations Children’s Fund (UNICEF 2013) estimates that between 5% and 10% of all children in Africa are children with disabilities where 90% of these children do not attend school and are thus less likely to engage in other opportunities for social participation (Global Partnership for Children 2012; Hansen, Siame & Van der Veen 2014). In Zimbabwe, the only extensive data on the incidence of disability is provided by the National Disability Survey conducted in 1981 as part of the programme for the International Year of Disabled Persons. In the survey, it was estimated that 276 300 people who constituted 4% of the population in Zimbabwe have moderate to severe disabilities.

The process of service development for people with disabilities in developing countries is called the community-based rehabilitation (CBR) (Rifkin & Kangere 2002). CBR was developed because institutional rehabilitation was not meeting the needs of persons with disabilities worldwide (Rifkin & Kangere 2002). In Zimbabwe, CBR was developed both by the government, with World Health Organization (WHO) and International Labour Organisation assistance, and by the civil society sector. The central thrust of the CBR programme spearheaded by the Ministry of Health (MOH) and Child Welfare has been the training of Rehabilitation Assistants to work at district hospitals and provide a referral chain, as needed, from community or-village level workers through to central hospitals and national rehabilitation centres. The main thinking behind this approach has been that the process of rehabilitation has to be broad-based, involve the grassroots and, above all, must be participatory and inclusive. In simple terms, there must be as much rehabilitation as possible, which is to take place in the home, at local clinics or at district hospital level.

In the Manicaland Province, World Vision has taken the lead in the adoption of CBR programmes. The programmes, which started in 2002, targeted the 6 wards (12, 16, 17, 18, 20 and 22) in Chipinge district where World Vision is operating (World Vision 2012). The adoption of CBR came as a result of failure to achieve impact of programmes that targeted children with disability that had been established prior to 2002. The programmes that targeted children with disability mainly dealt with medical and charity issues and ignored other important factors of life that lead to holistic achievement of quality of life for children. These include issues such as access to school, community’s negative attitudes towards children with disability and integration of these children in the community.

The focus then of the CBR programme in Chipinge district, which was being spearheaded by World Vision International, was on raising community awareness on children living with disabilities (CWD) and advocating against discrimination; integrating CWD in schools; supporting sports and events for CWD; providing household toilets for People Living with Disabilities (PWD) and school latrines for CWD and work with MOH, to introduce physiotherapy to CWD using locally available resources (World Vision 2012).

However, although the CBR programmes are being implemented in different countries and communities, less is known about its effectiveness on improving quality of life and disaster resilience for children with disability in rural Zimbabwe. This research therefore sought to investigate the effectiveness of the CBR project in Ward 20 of Chipinge district in Zimbabwe and ascertain the positive changes in the quality of life and disaster resilience of children with disability.

### Disability and disaster resilience

Resilience is a nebulous concept that has been adopted and adapted by numerous disciplines (Alexander 2013; Vale 2013). As a result there is no clear definition of what resilience is and the extent to which it can be applied. However, according to Mitchell and Harris (2012), recent literature have focused on resilience more as a process than an outcome, involving learning, adaptation, anticipation and improvement in basic structures, actors and functions. The UNISDR (2009) defines resilience as:

the ability of a system, community or society exposed to hazards to resist, absorb, accommodate to and recover from the effects of a hazard in a timely and efficient manner, including through the preservation and restoration of its essential basic structures and functions. (p. 24)

On the other hand, disability is a broad term that is not consistently defined. Its use varies according to societal norms and by the organisations, medical entities and governmental agencies that address disability issues (Peek & Stough 2010).

Handicap International (2009) emphasises impairment, barriers, accessibility and inclusion as key issues to do with disability and disasters. In Handicap’s submission, an impairment, defined as ‘problems in body function or structure such as a significant deviation or loss’, creates conditions of disability, seen as resulting ‘from the interaction between persons with impairments and attitudinal and environmental barriers that hinders their full and effective participation in society on an equal basis with others’ (Handicap International 2009:2). Accessibility, a central objective of mainstreaming disability in DRR, is defined as:

the characteristics of structural (such as buildings, roads, water supply systems) and non-structural items (such as information and communication systems) that enable their use by all members of a community, including those who have physical, sensory, mental or intellectual impairments and those who are older, younger, pregnant, unwell or injured. (p. 3)

Environmental and attitudinal barriers lead to exclusion and restriction in participation and representation, and this is what this CBR project in Chipinge attempted to dismantle. Barriers can be originating from the social environment such as political and legal environment and social cultural factors (e.g. belief systems) emphasised in this study. Barriers can also be originating from the physical environment, comprising the natural environment and built space. Thirdly, they can be linked to inaccessible information and communication, for example, early warning messages that do not accommodate people with disabilities.

Handicap International (2009) goes on to explain that PWDs are more vulnerable to disasters because of a number of factors. Firstly, PWDs are often part of the poorest groups of people and are among those most exposed and most vulnerable to extreme events (Peek & Stough 2010). Likewise, during emergencies the needs of individuals with disabilities are rarely addressed because of inadequate personnel and limited knowledge of disability (Peek & Stough 2010). Peek and Stough went on to explain that this could result in children with disabilities being left behind in an evacuation or forced to evacuate without supports. This has been further elaborated by The Global Partnership for Disability & Development and The World Bank (2009), cited in Sjuve (2015), who state that it is a common experience that in a disaster, emergency or conflict setting, PWDs are more likely to be left behind or abandoned during evacuation. Furthermore, disasters also lead to disabilities as they can create new impairments and new disabilities. For example, it is estimated that PWDs rose from 800 000 to 1.1 million after the 2010 Haiti earthquake where hundreds of children lost their limbs and many other were forced to undergo amputations as a result of secondary infections (Alexander 2011; Sjuve 2015).

### The essence of the community-based rehabilitation model

The theoretical conceptions and practices of CBR are based on a number of assumptions and principles. According to FSCE (2000), the primary principle of CBR model is encouraging participation of persons with disabilities and their families as a key factor in the rehabilitation process. FSCE (2000) argues that local participation is first needed because it allows mobilisation of local community for rehabilitation and development purposes. This means that participation enables growth of local capacity, which develops out of partnership between development agencies; community and disabled people themselves (FSCE 2000). Accordingly, the assumption is that working with target community instead of working for them is an essential strategy used in CBR model (FSCE 2000). This shows that deciding and doing things for persons with disabilities denies them the chance to learn and gain experience by making decisions.

According to O’Toole (1991), the key success of CBR is measured by active participation of the community. Thus, the principles of ‘involvement’ refer to making as many members of persons with disability as possible aware of their needs and stimulating their desire to do something about the needs (FSCE 2000). Further, the principle of ‘cooperation’ emphasises the need for collaboration of efforts between or among as many members of a group of a community as possible in resolution of their problems (FSCE 2000). In this regard, empowerment of persons with disabilities is very crucial. An empowered disabled person or group of persons with disabilities may be enough to bring to the attention of the community their needs and what should be done about them (WHO 1996a).

The other assumption relates to the resources needed for the improvement activities of persons with disabilities. The community mobilises and uses its own material, labour and monetary resources. The most important resources for CBR activities are the local community, which encompasses the people, the institutions, the materials and the money, which enable CBR to function and grow. These include human resources (persons with disabilities, parents, extended family, peers, professional people, religious and business people), institutional resources such as local schools, health clinics, vocational trainings and clubs and local natural resources, financial resources and other services (FSCE 2000).

### General understanding of rehabilitation for people with disability

Rehabilitation is aimed at helping children or people with disabilities to attain quality of life through enhancing the PWD natural abilities in the natural environments. The process of enhancing natural abilities in natural environments is described by the Rehabilitation Centre for Children as the type of service that assists CWD in their own communities by sharing information and transferring knowledge and skills to caregivers (WHO 1996a). This shows that rehabilitation that provides service in the CWD’s natural environment is very effective because the child is most likely to demonstrate his or her abilities in a familiar setting.

Measures used to assist PWD to improve their abilities in activities such as self-care, communication, moving around and developing vocational skills are generally considered to be rehabilitation measures (WHO 1996). Rehabilitation is also identified as (MOH RH Unit 1990):

the restoration of the handicapped to the maximum physical and mental utilization of which they are capable. (p. 27)

This implies that rehabilitation is generally considered to be the component of tertiary prevention that focuses on the reduction and elimination of a disability.

FSCE (2000) states that the third-level or territory intervention aims at creating equal opportunities and the full integration of PWD. This idea is supported by Helander (1999) who postulates that rehabilitation includes not only the training of disabled people but also interventions in the general system of society, adaptation of environment and protection of human rights. Rehabilitation is thus aimed at understanding PWD, their rights and special needs to empower them (Tirusew 1998).

Rehabilitation should be understood as a rights issue and not charity according to the current discourse in the development arena. The United Nations Declaration on the rights of disabled persons (UN 1975) emphasises that ‘Persons with disabilities have the right to services such as human and legal rights’. In addition to this, the Convention on the Rights of the Child (UN 1989) recognises children’s right to education and the right to rehabilitative services ensuring that children with disabilities have effective access to receive health, care services, education, training, preparation for employment, recreational opportunities and rehabilitation services in a manner conducive for the children’s achievement of the fullest possible for individual development and social integration. The convention on the right of the child was ratified by the Zimbabwe government in 1990 and it infers that people with disabilities in general have the need and rights to all aspects of rehabilitation, that is, social, educational, medical and psychological in order to maximise their potential and minimise disability.

Helander (1999) points out that disability imposes a considerable social, economic and emotional cost on disabled people, their families and wider community. This means that if there is no effective rehabilitation, disabled people, particularly CWD, may lead unhappy, dependent lives and become burdens to their families and to society.

CBR is arguably therefore a form of adaptation to the challenges brought about by society on PWDs. In CBR, interventions were to be shifted from institutions to the homes and communities of people with disabilities and carried out by minimally-trained people such as families and other community members, thereby reducing the financial costs.

In the past, CBR approach was viewed as an alternative to the institutional model of rehabilitation. This has changed with CBR now viewed as an approach that provides a link between the community worker and the professionals, and link between the disciplines of health and education (Fulcher 1989).

CBR and Institutional Based Rehabilitation (IBR) could have many major differences though they complement each other. The CBR philosophy advocates are discouraging the policy of bringing groups of disabled to centres, but encouraging the institutions to move to the beneficiaries. The ideal would be to have movement both ways, with institutions running outreach services in local communities where it is feasible to do so, and clients travelling in to the institutions for the special services that could not still be provided locally (United Nations Educational Scientific and Cultural Organisation [UNESCO] 1994, cited in FSCE 2000). As this assumption indicates, the involvement of institutions in community rehabilitation services is very vital together with CBR services.

### Understanding quality of life

In the Consensus Document prepared by the International Association for the Scientific Study of Intellectual Disabilities for WHO in August 2000, the core ideas that have emerged in the international literature are summarised and a framework for how quality of life could be understood and measured is provided. Quality of life is described as an aggregate of different, interrelated aspects of life. These aspects can be organised into domains. In this document, 8 domains of well-being are suggested:

- *Inter- and intra-personal variability*: Variability means that the domains of well-being will apply to or be experienced by individuals and cultural groups to varying degrees. A good quality of life means different things to different people.
- *Personal context*: People are best understood within the context and environment important to them – where they live, work and play. Environments should be adaptable so as to accommodate personal interests and needs.
- *Life span perspective*: Quality of life includes a life span perspective. Support and services denied during childhood can affect quality of life later in life and thus have cumulative effects.
- *Holism*: This means that all the domains of well-being are interrelated. Particular aspects or domains of an individual’s life may dramatically influence other domains.
- *Values, choices and personal control*: Quality of life recognises different value systems and accepts that choices and personal control over activities, interventions and environment have major implications for self-image, motivation, self-expression and health.
- *Perception*: An individual’s perception about his or her own quality of life is important. There is no correct or incorrect response. Sometimes it is important to take into account the perceptions of parents, spouses or service providers. However, it should be noted that these perceptions might differ appreciably from the perceptions of the individual.
- *Self-image*: The aims of all quality of life programmes must be to enhance the individual’s self-image and to provide empowering environments, which enhance opportunities to control the personal aspects of life.
- *Empowerment*: Quality of life assumes major control by the service user over services provided and interventions designed. Detailed examination is needed to discover who controls the programmes and interventions to ensure that this can happen.

The Consensus Document concludes that there are both objective and subjective quality of life indicators. Objective indicators can be reliably observed and measured, for example, material attainment, stability of human institutions, social connections and life opportunities. Such indicators can be measured in quantitative studies. Subjective indicators measure quality of life, as it is understood and valued from the individual perspective, and identify those specific aspects that become valued by individuals as they pursue their lives in their unique environments. These indicators are identified by qualitative studies. This study focused on the subjective, qualitative indicators.

### Situating the study

Chipinge World Vision CBR programme covers 6 wards, namely 12, 16, 17, 18, 20 and 22 but this study focused on Ward 20. The rationale for selection of this ward was because it was the only ward of the World Vision programme where CBR programming was implemented, on the basis of initial programme assessments. Convenient sampling was used to select Ward 20 as one of the researchers grew up in the area and gaining access to the gate keepers became easier. The study focused on children in Chipinge who had various forms of disabilities such as physical disability, hearing and speech impairment and visual impairment.

Chipinge district falls under Manicaland Province, which is often fraught with extreme drought in some parts of the province. Flooding risk is low, but can be very high because of cyclones that are mostly driven by its proximity to Mozambique, a country affected by the oceanic wind systems. Mutare, Makoni, Buhera, Chipinge and Chimanimani districts are the hardest hit by drought when it occurs, while Chipinge South is more prone to floods. Bongo (2015) submits that Manicaland is also affected by high level of erosion, mostly soil erosion. This fuels a lot of impacts like siltation of rivers and other water reservoirs, affecting the overall availability of water for agricultural and domestic purposes, which in turn affects agriculture, food security and nutrition. This provides the brief background of disaster risk in the study area.

## Methodology

This study focused on children in Chipinge Ward 20 who have various forms of disabilities such as physical disability, hearing and speech impairment and visual impairment. The study adopted qualitative research design because of the type of data that this research intended to generate. The information that the research generated was non-statistical but sought to explain human behaviour in relation to their environment. Semi-structured interviews, focus group discussions, key informant interviews and document analysis were used to assess the effectiveness of CBR programmes in improving the quality of life and disaster resilience for children with disabilities. Researchers selected 4 groups of people where we wanted to sample. These included children with disabilities who were part of the CBR programme, parents and guardians of the children with disabilities, non-profit organisations (NGOs) based in Chipinge working with children with disabilities, and disaster management and local government members who work within CBR and child welfare issues. As a result, the sample respondents comprised 8 parents of children with disabilities, 8 children with disabilities, 1 World Vision official, 2 village heads, the ward councillor, Village Health worker and 1 other informant from government institutions, making a total of 22 participants.

Purposive sampling methodology was used where participants were intentionally selected to reflect particular features, or to represent certain groups. In this case, children with disabilities and their parents and all institutions that had a part to play in CBR were selected. Only those children who were under the World Vision International CBR programme participated. Semi-structured interviews were conducted with 8 parents of 8 children with disabilities. All research procedures were communicated to participants and their concerns were listened to. Participants were advised of their rights to withdraw or refuse to participate in the research at any stage. A total of 6 key informant interviews were also conducted. Interviews with key informants were held at places convenient to each interviewee, and each interview took about 30 min.

One focus group discussion with 8 parents, an NGO official and a councillor was held. Focus group discussion allowed the researchers to collect information from all key people working with children with disabilities drawn from different areas. Focus group interview explored the effectiveness of CBR and the challenges faced. Participants discussed the questions, helping each other with the answers. At times they reminded each other about details. They also managed to ask each other additional questions which were recorded to provide supplemental data. Finally, document analysis was also used especially reports from the NGOs that were working with children with disabilities. All data were transcribed and analysed manually.

## Results

### Contribution of community and parents of children living with disabilities in initial design and implementation of the programme

The research found out that there was little involvement of the general community and parents of CWDs in the initial design and implementation of the programmes. Discussions with key informants indicated that the CBR programme in Chipinge district in general was a brain child of the Ministry of Health Rehabilitation Department after realisation that PWDs were not accessing medical care and other services. MOH then approached NGOs such as World Vision and Plan International for support in the initial design and implementation of the programme.

Focus group discussions with the community members in Nyerere Village also indicated that the CBR programme was actually handed over to them by government stakeholders and NGOs. One respondent in the focus group discussion (FGD) said:

‘Chirongwa chekuti vana vakaremara vaonekwe kuno kumaruwa takachiturirwa nevehurumende.’ [The programme of community based rehabilitation was brought to us by the government.] (Local leader, female in her early 50s)

The FGD with parents of children with disability revealed that it was World Vision and the MOH that introduced them to CBR while the FGD for children had little to say on the subject as they were not sure how the programme started. One informant from Machena Village said:

‘Ndakangoonawo vanhu vachiuya kumba kuzondipa wheelchair nekundiitisa maexersise.’ [I just saw people coming to our house to give me a wheel chair and conduct physiotherapy.] (Boy child with disability, 10 years old)

While the community and PWDs had no input in the initial design of the programme, it was evident that they became involved at a later stage. The Rehabilitation Officer from the MOH then trained family members on home-based physiotherapy and general care of children with disability. Community members also contributed labour and local resources for construction of Blair toilets with rumps at schools and at their homes. This information was provided by key informants and it also came out during the FGDs that were conducted with CWD, community members and parents of CWDs.

Table 1 shows results of the household survey on community education on rights of CWD. All parents and children participants identified schools (teachers) as the major source of information on rights of children with disability for CBR (29%) followed by village health workers (24%) and community leaders (15%). This shows that that there is a degree of involvement of community members during the implementation of programmes as they participate in educating other members of community on issues of disability.

**TABLE 1:** Sources of information on rights of children with disability.

Information source	Frequency	%
NGO	1	0.89
Churches	5	4.46
Health centre	3	2.68
Community leader	17	15.18
Health worker	27	24.11
Parents	5	4.46
School	33	29.46
N/A	21	18.75

**Total**	**112**	**100.00**

Note: When asked to indicate the sources of information and knowledge on rights of children with disability, the highest frequency was the school, followed by health worker and community leader. This shows the important role played by local institutions in disseminating information on the rights of children with disability.

NGO, non-profit organisations; N/A, Not Available, for respondents who did not have any answer on the question.

Tenants of the CBR programme postulate that for CBR to be effective, the community and PWDs have to meaningfully participate in the design and implementation of the programme (O’Toole 1991). The findings of this research show some community participation as described above but the community still shows their participation is limited to providing labour and receiving information on how to occasionally do physiotherapy for their children at home. There is still high dependency on donors and government departments who ‘brought’ the programme to them. This is supported by the fact that the household survey responses also indicated that the CBR programme would not continue if World Vision, who is the major financial contributor, has left the community.

### Benefits of community-based rehabilitation for children with disability

Parents and children with disabilities cited provision of school fees and food, psychosocial support and clothes as the major benefits that CWD got from the CBR programme. These results were also confirmed when one key informant was saying:

‘All CWDs received a lot of support from the NGOs. They are given food supplies, clothing and their school fees is paid and other services.’ (Local teacher, male in his early 40s)

Children living with disabilities and their parents in the study reported that CBR had helped them to manage disability in that some parents and families were involved in day-to-day physiotherapy exercises with their children. Children reported that CBR had helped them to gain some knowledge about their disability and receive meaningful help from their families. This help included being escorted to school, creating space for CWD during community games and other interactive processes and improved assistance with going to the toilet, among other things. They also pointed out that participation of children in CBR activities has enhanced their self-confidence and reduced feelings of isolation and dependency. This was particularly true for those children who had received assistive devices such as wheelchairs. During a focus group discussion, a 14-year-old child who cannot walk and received a wheelchair said:

‘Kubvira pandakapihwa wheel chair kufamba kwakundiitira nani chero hazvo wheel chair yacho ichinetsa kufambisa ndoga munzvimbo dzine jecha.’ [Since I received a wheel chair mobility is now easier even though it is difficult to move the wheel chair in sandy places.]

In relation to this, it was reported that participation in rehabilitation process helped children transform their prior passive role to active and valued roles in the community, for example, some children were able to participate in sporting activities at school. This is supported by one of the children who said: ‘I go to school and I was told that I can do all things just like any other child. I can read and write and play soccer’ (boy with disability, aged 12).

An annual report from World Vision (2012) and records from 2 primary schools in the ward indicate that the former financially supported Ministry of Education carries out assessment of children with special needs. Two special classes established at these schools received special learning aids such as books and toys.

### Attitudes towards children with disability as a result of community-based rehabilitation implementation

Questions asked to determine issues of attitude change in the household survey show that community perceptions towards CWD are to a greater extent positive. In responding to questions on whether they would send a child with disability to school or care for them in their household, respondents gave positive feedback. All the 8 parents said they would send CWD to school as well as care for them in their home. A great number of the respondents also indicated that they did not believe that CWD should receive treatment from traditional healers or prophets.

The interview with the Rehabilitation Officer revealed that although there were notable positive changes realised in the way PWDs were perceived in Ward 20 after CBR implementation, transformation of the mind was a process. A lot of children who had been labelled ‘zvikwambo’ (Goblins) and kept closed-in in homes had been enrolled in the CBR programme. These mainly included those who had cerebral palsy because the community did not understand that this was a physical disability. The Social Services Officer confirmed that the practice of hiding CWDs is slowly dying in the area because they had only handled one such case in the last 5 years compared to before the intervention where they would attend to between 2 and 3 cases that would have been reported per year. In terms of changing the negative attitudes against children with disability, the research showed that the CBR programme in Ward 20 of Chipinge is being effective. Changes in attitude help CWDs in that they can freely participate in all activities that other children are involved in without stigma and discrimination. The Schools Psychological Services Officer said that there was a marked improvement in the enrolment of CWDs since the inception of CBR. Principles of inclusion of marginalised people are thus being realised through attitude change in Chipinge. These findings to a larger extent confirm the ideas by Mannam and Turnbull (2007) that CBR should focus on contextual factors such as helping non-disabled persons in the community accept people with disabilities and promoting social integration for people with disability.

### Effectiveness in usage of local capacities and resources in the community-based rehabilitation programme

The research found out that the CBR programme in Ward 20 has strength in that it is being run by government workers who reside in the area. World Vision only provides financial resources while government employees provide the necessary technical skills. These include a Rehabilitation Technician, the Social Services Officer and the Schools Psychological Officer from Chipinge district. The Rehabilitation Officer is responsible for training families and children in physiotherapy in their homes and disseminating information on the causes of disability in the community. The Social Services Officer is responsible for the general welfare of children with disability and all programmes that are done for PWDs are reported to him. His duties also involve psychosocial support for PWD and their families. The Schools Psychological Officer is responsible for ensuring that CWD have access to school and she makes any referrals to special schools.

The Rehabilitation Officer indicated that MOH had conducted training for at least 65% of families with CWD to conduct simple physiotherapy sessions in the home environment. This practice helps to improve quality of life for CWD because instead of walking long distances to health centres to access services, they access the services in the comfort of their homes. It is also cheaper to conduct the sessions at home because they do not pay clinic fees. The findings above affirm that CBR is a process of enhancing natural abilities in natural environments (WHO 1996). CWD are assisted in their own communities by sharing information and transferring knowledge and skills to caregivers. The use of locally available resources such as water, sand, labour and timber in construction of user-friendly toilets and ramps in schools and homes were mentioned in this study.

As shown in Table 2, majority of parents and children who participated in the study confirmed that community leaders were very much involved in the CBR programme. This was also confirmed during the FGD which indicated that community leaders were very instrumental in issues of disability, especially on mobilisation, and some of them were part of those that were carrying out education on disability. The FGD revealed that the involvement of local leaders was instrumental in that these were opinion leaders who were respected in the community. They act as the custodians of knowledge in the area. Parents and families of CWD are also being involved in the physiotherapy sessions for children.

**TABLE 2:** Involvement of community leaders.

Extent of involvement of leaders in the WV Rehabilitation Programme	Frequency	Percentage
Very involved	38	32.2
Involved	58	49.2
Not sure	2	1.7
Not involved	6	5.1
Not applicable	14	11.9

**Total**	**118**	**100.0**

Note: Participants in the study were asked to comment on the extent of involvement of local leaders in the WV community-based rehabilitation programme. Of the respondents, 32% said that local leaders were highly involved, while 49% indicated that they were involved. Only 5% indicated that local leaders were not involved. This high level of involvement of local leaders contributed towards achievement of positive outcomes for the programme.

West Virginia (WV) Rehabilitation programme, Chipinge community-based rehabilitation programme.

Despite the contribution that local capacities and available resources have made to CBR programmes key informants maintained that these were not enough to drive the CBR programme. The interviews made with them revealed that the government and Chipinge Rural District Council need to commit financial resources to complement community efforts.

### Gaps limiting the effectiveness of the community-based rehabilitation programme

The key informant interviews revealed some obstacles that hinder the implementation of CBR programme. Discussions focused on the ways and means of identifying obstacles in order to turn them into opportunities for growth. The main challenge was the lack of commitment of resources by government and Chipinge Rural District Council to issues of disability. One of the respondents said that since the inception of the CBR programme in Chipinge district, the council had not allocated ‘even a cent’ to the issues of disability in Chipinge district. He said while a lot of effort is being put in the CBR programme it is sad that once the big donors leave there will be a yawning gap in terms of provision of resources for CBR. This is in line with findings of The International Consultation to Review CBR (WHO 2003) which stated that the problem with sustaining CBR programmes in many countries, including those in Africa, had to do with inappropriate approaches whereby some programmes were initiated and operated as separate or add-on programmes. The review indicated that CBR programmes should be tailored towards integration into mainstream services and funding structures. The same report also observed that strictly bottom-up programmes were often not sustainable because they tended to alienate governments or were unlikely to receive full support and involvement of government.

The referral system for access to services for CWDs was weakened by poverty and lack of resources. In the education sector, for example, the Social Services Officer and the Schools Psychological Officer said that in instances where children needed to be referred to other schools because of their special needs, there was a challenge in getting the required funds. An example that was given during the interviews was that Chipinge Primary School, which has facilities for special education, is inaccessible to CWD because it offers boarding facilities which are not covered under the Basic Education Assistance Module. Referrals to schools outside Chipinge such as Jairos Jiri are also very difficult as families have to foot the transport bills to accompany their children to these schools. The bus warrants that the Department of Social Services relied on in such instances are no longer being accepted by transport companies because the department is facing financial challenges thus failing to meet their obligations to suppliers.

## Discussion

The goal of CBR is to influence people, their perception, attitudes and behaviour. In this study, the focus was on special needs of children with disability as a point of entry. Thus, CBR has been successful in reaching some of the poorest families and their children with disabilities by making daily life better with valuable, practical skills, devices, creating integration and lessening discrimination and social integration in Ward 20 of Chipinge district. Community and parents’ awareness of the causes of disability is significantly changed from religious beliefs to other possible causes as a result of CBR programme intervention. This is true because in the process of transferring knowledge and skill, CBR workers have promoted positive emotional, social and cultural values to the existing realities. The community in Chipinge has developed positive expectations to children with disability with regard to their educational performance and active participation at family and community levels. In mobilising and using local resources needed for rehabilitation of children with disabilities, the CBR programme under study showed minimal efforts in fund raising locally. Except for labour and other natural resources found in the area, the programme was highly dependent on external funds.

### Participation of community and participation in community-based rehabilitation

From the study, majority of the people in Ward 20 of Chipinge district view CBR as an effective and important tool for improving quality of life for CWD. Despite this support for CBR, the study also showed that there are still challenges relating to meaningful participation of the community and parents of CWD in design and implementation of the programme. Participation in CBR programmes is still limited to less important roles such as contributing labour and receiving information on how to conduct physiotherapy sessions at home. The more decisive roles in the implementation of the CBR programme still remain with government stakeholders and members of the civil society. The effectiveness of CBR is inseparable from an analysis of the context and environment in which implementation of the programme takes place. This therefore means that definitions of components of CBR such as community participation, local resource mobilisation and inclusivity vary depending on the context. Issues of societal values and competing development priorities have to be taken into consideration.

### Resource allocation for community-based rehabilitation

While the concept of CBR appears to be the panacea for achieving quality of life for CWD in developing countries, the major hindrance to its success has been lack of commitment of resources by government and local authorities to the programme. CBR has thus relied on donor funds which are short-term and inconsistent. There are fears that if there is no support from the civil society sector, the continuity of the programme is doubtful.

### Attitudes towards children living with disabilities

The CBR programme can effectively lead to a change in attitudes towards children with disability. This was demonstrated in the study by the fact that the since the introduction of the CBR programme practices such as hiding disabled children in Ward 20 of Chipinge district slowly declined. The study also showed that attitude change is a process not an event as there are still minimal reports of some parents hiding children with disability. Some community members also still believe that disability should be dealt with by prophets or traditional healers.

### Use of local capacities and resources in community-based rehabilitation programmes

The effectiveness of CBR requires collaborated efforts from different stakeholders. This was exemplified in the study by efforts between government departments such as MOH, Social Services and Ministry of Education who worked together with the communities and civil society to achieve holistic quality of life for CWD. There is a challenge however in that the local resources contributed by the community such as labour and building materials for toilets and ramps though important cannot sustain the CBR programme. Rehabilitation for CWD is a highly specialised area that requires special equipment that is not locally available. Financial resource mobilisation from poor communities is also difficult considering that there are other competing priorities that the communities have.

### Contribution of community-based rehabilitation to achieving quality of life for children living with disabilities

CBR is an important strategy in reaching out to CWD in that the community and government become more aware of the needs of CWD. This leads to improvements in the way infrastructure is constructed to be user-friendly for CWD. There are also deliberate efforts to pool resources together for provision of assistive devices, resource units in schools that provide for the needs of CWDs. Changes in community perceptions for CWD also contribute to better quality of life for CWD in that communities ensure that CWD have access to basic services. These include education and health.

### Community-based rehabilitation and resilience in relation to Sen’s capability approach and Chambers’ bottom-up approach

Sen’s theory deals with the structures causing inequalities and poverty. He views the individuals as active subjects, agents with capabilities. These capabilities can be promoted or hampered by the policy in the broader society. Sen (1999) presents 5 distinct freedoms: political freedoms, economic facilities, social opportunities, transparency guarantees and protective security. Each freedom increases the general capability of a person and interacts to reinforce other freedoms. They promote basic human or substantive freedoms that include elementary capabilities like being literate, avoiding undernourishment, et cetera. The expansions of freedoms are viewed not only as the ‘principal means’ but also as the ‘primary ends’ of development (Sen 1999:10, 36).

Amartya Sen’s theory combines with the approach of Robert Chambers, who in his books *Rural Development – Putting the Last First* (1983) and *Whose Reality Counts? – Putting the first last* (1997) presents a democratic participating perspective. In the ‘deprivation trap’, he concludes some of the complexity regarding poverty, the elements there described consist of powerlessness, isolation, vulnerability, physical weakness and poverty. These elements affect each other, depriving the capabilities of the persons (Chambers 1983, 1997). According to Amartya Sen, individual capabilities depend not only on the individual but among other things on ‘economic, social and political arrangements’ (Sen 1999). He joins with Chambers in emphasising the importance of the democratic inclusive processes, where:

people have to be seen, in this perspective, as being actively involved – given the opportunity – in shaping their own destiny, and not just as passive recipients of the fruits of cunning development programs. (Sen 1999:53)

According to Thomas (2005), disabled people share the profile of the general poor, but they experience poverty more intensely and have fewer opportunities to escape poverty than the non-disabled. They largely remain trapped in a vicious circle of poverty and social exclusion. Underestimated and undervalued by others, they begin to doubt their own abilities, and the image of the disabled person as a passive victim becomes a self-fulfilling prophecy. This corroborates Sen and Chambers’ submissions on eroded instrumental freedoms and capabilities and the influence of the ‘deprivation trap’ on CWD’s capacity to engage in livelihood and disaster risk reduction (DRR) initiatives. In the same vein, on the basis of field findings presented above and the background information, it is imperative to mainstream disability in disaster risk reduction and development programming in general for the following reasons, as suggested by Handicap International (2009:26). The first reason is the link between disability and poverty: PWDs and households with PWDs often belong to the poorest. Hence, they are particularly vulnerable (e.g. living in hazardous areas in shelters of poor quality) and have less capacities to deal with disasters (e.g. less financial means to face increasing food prices). Secondly, the link between disability and disasters: PWDs are more vulnerable in disasters because of their impairment, existing barriers and their socio-economic situation. Thirdly, disasters create impairments or disability. Fourthly, PWDs tend to be invisible in disasters. Aid often focuses on people who became impaired through the disaster, neglecting people who were already impaired. Fifth, not including PWDs in DRR means putting new barriers for them and not letting them participate in development processes. Finally, when the disaster strikes, time is often running: lack of time combined with lack of knowhow and negative attitudes might cause obstacles to mainstreaming. The right to live, the right for shelter, et cetera, are human rights equal for everybody. It is therefore an obligation to include PWDs in disaster response. If action is taken prior to a disaster, chances for a comprehensive inclusion are clearly higher.

Stintzing (2006) describes in her study ‘Being parents/guardians of disabled children in a low-income area in rural Mexico’ how parents to disabled children ‘withdrew socially’ as they felt ‘not being good enough’. Some had experienced stigmatisation and multiple discrimination ‘for being indigenous people, poor, women, parents of a disabled child, all the classical marks of stigma’ (Stintzing 2006, cited in Davidsson, 2010:9). Aaron Antonovsky (1991) contributed to explain the empowering process with his theory concerning *sense of coherence*. The sense of coherence is built up by an ability to *understand* one’s situation and what happens, the resources needed to be able to *handle* or deal with it and finally experience a feeling of *meaningfulness*. The increased understanding, ability to handle and feeling of meaningfulness is an empowering process contributing to a sense of coherence which increases the possibilities to cope with challenges in life.

In developing countries, fundamental material improvement may be relevant for parents to participate in interventions that target their children. Poverty, severe social stress, unemployment, overwork and tension hamper parental involvement in rehabilitation process. In such cases, the disabled child’s progress or determination in all aspects may be the least of the parent’s worries for practical reasons of unemployment or poverty or negative attitudes.

## Conclusion

According to a joint position paper by ILO, UNESCO and WHO (2004), CBR is meant to demystify the rehabilitation process and give responsibility back to the individual, family and community. This idea implies that CBR should be perceived as part of community development where the community is mandated with dealing with issues of rehabilitation for PWDs. Helander et al. (1989) affirm the idea and say once the community takes on the responsibility of rehabilitation for persons with disabilities, then the process could truly be called CBR. In this case, therefore, rehabilitation becomes one element of a broader community integration effort.

In light of the arguments raised in the study, it can be noted that CBR programmes can only be effective if there is deliberate effort to involve the local community and parents of CWD in the implementation and design of programmes. Negative perceptions of disability also have a negative effect on the achievement quality of life for children with disabilities and any effective CBR programme should focus on this aspect. The issue of local resource mobilisation for CBR programmes against other competing priorities in a community with limited resources was also an important area of focus for this study. This is because issues of disability may not be taken as a priority as they affect a marginal group of the population, with Ward 20 constantly facing other challenges such as droughts, floods and cholera outbreaks which affect larger populations.

## References

[CIT0001] Alexander D.E., 2011, ‘Disability and disaster’, in Wisner B., Gaillard J.-C. & Kelman I. (eds.), *Handbook of hazards and disaster risk reduction*, pp. 384–394, Routledge, London.

[CIT0002] Alexander D.E., 2013, ‘Resilience and disaster risk reduction: An etymological journey’, *Natural Hazards and Earth System Sciences* 1, 1257–1284.

[CIT0003] Antonovsky A., 1991, *Hälsans mysterium*, Natur och Kultur, Natur & Kultur Akademisk, Stockholm, Sweden.

[CIT0004] Bongo P.P., 2015, *Draft national agricultural hazard risk profile of Zimbabwe*, Research document prepared for the UN Food and Agriculture Organisation (FAO), Harare.

[CIT0005] Busapathumrong P., 2013, ‘Disaster management: Vulnerability and resilience in disaster recovery in Thailand’, *Journal of Social Work in Disability & Rehabilitation* 12(1–2), 67–83. https://doi.org/10.1080/1536710X.2013.784176 23679805 10.1080/1536710X.2013.784176

[CIT0006] Chambers R., 1983, *Rural development: Putting the last first*, Hong Kong, London.

[CIT0007] Chambers R., 1997, *Whose reality counts? Putting the first last*, Intermediate Press, London.

[CIT0008] Davidsson J., 2010, *Community-Based Inclusive Development as a strategy for Millennium Development Goals*, Bachelor Thesis, Uppsala University.

[CIT0009] Forum on Street Children Ethiopia (FSCE), 2000, *Investigating the intervention of community based rehabilitation program for children with physical disabilities in Adama Town*, FSCE, Addis Ababa.

[CIT0010] Fulcher G., 1989, *Disabling policies: A comparative approach to education policy and disability*, Falmer, London.

[CIT0011] Global Partnership for Children, 2012, *2012 forum*, viewed 30 September 2016, from http://www.unicef.org/disabilities/index_65775.html

[CIT0012] The Global Partnership for Disability & Development (GPDD) and The World Bank (Human Development Network – Social Protection/Disability & Development Team, 2009, *The Impact of Climate Change on People with Disabilities Report of the discussion*, viewed 05 July 2016, from http://www.cbm.org/article/downloads/82788/Ediscussion_on_climate_change_and_disability.pdf

[CIT0013] Handicap International, 2009, *Mainstreaming disability into disaster risk reduction: A training manual*, Handicap International and European Commission Humanitarian Aid, Lyon, France.

[CIT0014] Hansen A.M.W., Siame M. & Van der Veen J., 2014, ‘A qualitative study: Barriers and support for participation for children with disabilities’, *African Journal of Disability* 3(1), 112 https://doi.org/10.4102/ajod.v3i1.112 28730000 10.4102/ajod.v3i1.112 PMC5433439

[CIT0015] Helander E., 1999, *Prejudice and dignity: An introduction to community-based rehabilitation*, 2nd edn., United Nations Development Programme, Originally published 1993, viewed 15 July 2016, from www.einarhelander.com/PD-overview.pdf

[CIT0016] Helander E., Mendis P., Nelson G. & Goerdt A., 1989, *Training in the community for people with disabilities*, World Health Organization Library, Geneva.

[CIT0017] ILO, UNESCO & WHO, 2004, *Community based rehabilitation for and with people with disabilities*, Joint position paper, WHO Library, Geneva.

[CIT0018] Jackson H., 1993, *Challenging disability. A guide for frontline social workers in Africa (Module 6 - Social work and children with disabilities)*. Harare: School of Soc. Work, & Geneva: ILO.

[CIT0019] Jackson H. & Mupedziswa R., 1998, ‘Disability and rehabilitation: Beliefs and attitudes among rural disabled people on a community based rehabilitation scheme in Zimbabwe’, *Journal of Social Development in Africa* 3(1), 21–30.

[CIT0020] Mannan H. & Turnbull A.P., 2007, ‘A review of community based rehabilitation evaluations: Quality of life as an outcome measure for future evaluations’, *Asia Pacific Disability Rehabilitation Journal*, viewed 23 August 2016, from http://www.dinf.ne.jp/doc/english/asia/resource/apdrj/v182007/developmental_articles02.html

[CIT0021] Mitchell T. & Harris K., 2012, *Resilience: A risk management approach*, ODI Background Note, Overseas Development Institute, London.

[CIT0022] Ministry of Health Rehabilitation (MOH RH) Unit, 1990, *A report on eight rehabilitation projects*, Government Printers, Harare, Zimbabwe.

[CIT0023] O’Toole B., 1991, *Guide to community based rehabilitation services*, UNESCO, Paris.

[CIT0024] Peek L. & Stough L.M., 2010, ‘Children with disabilities in the context of disaster: A social vulnerability perspective’, *Child Development* 81(4), 1260–1270. https://doi.org/10.1111/j.1467-8624.2010.01466.x 20636694 10.1111/j.1467-8624.2010.01466.x

[CIT0025] Rifkin S.B. & Kangere M., 2002, ‘What is participation?’, in *Community-based rehabilitation (CBR) as a participatory strategy in Africa*, pp. 37–49, viewed 02 August 2017, from http://digitalcommons.ilr.cornell.edu/gladnetcollect/60%5Cnhttp://www.asksource.info/cbr-book/cbr03.pdf

[CIT0026] Sen A., 1999, ‘Introduction’, in Sen A. (ed.), *Development as freedom*, n.p., Oxford University Press, Oxford.

[CIT0027] Sjuve M., 2015, ‘Natural disasters impact on children, with a particular emphasis on girls and children with disabilities a case study from Haiti assessing children’s vulnerabilities to natural disasters and its underlying causes’, Masters degree thesis, University of Agder.

[CIT0028] Stintzing K., 2006, ‘Being parent/guardians of disabled children in a low income area in rural Mexico – A qualitative interview study’, Master programme, Department of Women’s and Children’s Health, Uppsala University.

[CIT0029] Thomas P., 2005, *Poverty reduction and development in Cambodia: Enabling disabled people play a role, disability knowledge and research*, viewed 25 November 2017, from http://citeseerx.ist.psu.edu/viewdoc/download?doi=10.1.1.882.7219&rep=rep1&type=pdf

[CIT0030] Tirusew T., 1998, *Psychosocial and learning aspects of disability and intervention strategies*, Department of Educational Psychology, Addis Ababa University, Addis Ababa.

[CIT0031] United Nations Children’s Fund, 2013, *Monitoring the situation of children and women 2012*, ChildInfo, Boulder, CO.

[CIT0032] United Nations (UN), 1975, *Declaration on the Rights of Disabled Persons Proclaimed by General Assembly resolution 3447 (XXX) of 9 December 1975*, United Nations, viewed 20 January 2017, from http://www.equalrightstrust.org/ertdocumentbank//DisabilityDoc.pdf

[CIT0033] United Nations (UN), 1989, *Convention on the Rights of the Child Adopted and opened for signature, ratification and accession by General Assembly resolution 44/25 of 20 November 1989*, United Nations, Geneva.

[CIT0034] UNISDR, 2009, *2009 UNISDR terminology on disaster risk reduction*, Geneva, viewed 15 August 2016, from http://www.unisdr.org/files/7817_UNISDRTerminologyEnglish.pdf

[CIT0035] Vale L.J., 2013, ‘Resilient cities: Whose resilience? Whose city?’, *Building Research & Information* 42(2), 191–201.

[CIT0036] World Health Organization (WHO), 1996a, *Disability prevention and rehabilitation*, WHO Library, Geneva.

[CIT0037] World Health Organization (WHO), 1996b, *Guideline for educating, monitoring, and self-assessment of CBR program*, WHO Library, Geneva.

[CIT0038] World Health Organization (WHO), 2003, *International consultation to review community based rehabilitation (CBR)*, WHO, Geneva.

[CIT0039] World Vision, 2012, *World vision chipinge ADP annual report*, World Vision, Harare, Zimbabwe.

